# Adaptation of H9N2 AIV in guinea pigs enables efficient transmission by direct contact and inefficient transmission by respiratory droplets

**DOI:** 10.1038/srep15928

**Published:** 2015-11-10

**Authors:** Xiaoyu Sang, Airong Wang, Jie Ding, Huihui Kong, Xiaolong Gao, Lin Li, Tongjie Chai, Yuanguo Li, Kun Zhang, Chengyu Wang, Zhonghai Wan, Geng Huang, Tiecheng Wang, Na Feng, Xuexing Zheng, Hualei Wang, Yongkun Zhao, Songtao Yang, Jun Qian, Guixue Hu, Yuwei Gao, Xianzhu Xia

**Affiliations:** 1 Harbin Veterinary Research Institute, Chinese Academy of Agricultural Sciences, Harbin, 150001, China; 2 Key Laboratory of Jilin Province for Zoonosis Prevention and Control, Military Veterinary Research Institute, Academy of Military Medical Sciences, Changchun, 130122, China; 3 College of Animal Science and Veterinary Medicine, Shenyang Agricultural University, Shenyang, 110866, China; 4 College of Animal Science and Veterinary Medicine, Shandong Agricultural University, Tai'an, 271000, China; 5 College of Animal Science and Technology, Jilin Agricultural University, Changchun, 130000, China; 6 Changchun Veterinary Research Institute, Chinese Academy of Agricultural Sciences, Changchun, 130122, China; 7 Jiangsu Co-innovation Center for Prevention and Control of Important Animal Infectious Diseases and Zoonoses, Yangzhou, 225000, China

## Abstract

H9N2 avian influenza viruses circulate worldwide in poultry and have sporadically infected humans, raising concern whether H9N2 viruses have pandemic potential. Here, we use a guinea pig model to examine whether serial passage results in adaptive viral changes that confer a transmissible phenotype to a wild-type H9N2 virus. After nine serial passages of an H9N2 virus through guinea pigs, productive transmission by direct contact occurred in 2/3 guinea pig pairs. The efficiency of transmission by direct contact increased following the fifteenth passage and occurred in 3/3 guinea pig pairs. In contrast, airborne transmission of the passaged virus was less efficient and occurred in 1/6 guinea pig pairs and 0/6 ferret pairs after the fifteenth passage. Three amino acid substitutions, HA1-Q227P, HA2-D46E, and NP-E434K, were sufficient for contact transmission in guinea pigs (2/3 pairs). The two HA amino acid substitutions enhanced receptor binding to α2,3-linked sialic acid receptors. Additionally, the HA2-D46E substitution increased virus thermostability whereas the NP-E434K mutation enhanced viral RNA polymerase activity *in vitro*. Our findings suggest that adaptive changes that enhance viral receptor binding, thermostability, and replicative capacity in mammalian cells can collectively enhance the transmissibility of H9N2 AIVs by direct contact in the guinea pig model.

Influenza viruses pose a continual threat to public health in the form of seasonal epidemics and occasional pandemics. To date, influenza viruses bearing H1, H2, H3, H5, H6, H7, H9 and H10 hemagglutinin (HA) subtypes have infected humans^1^,^2^,^3^,^4^,^5^. Of these, only three HA subtypes (H1-H3) have established stable lineages in humans. Although rare, the H9N2 avian influenza virus (AIV) subtype has been reported to cause human infections^2^,^6^. H9N2 AIV was first detected in poultry in the United States in 1966^7^ and has circulated as the most prevalent influenza virus subtype in poultry in China since 1994^8^. Serological surveillance for H9N2 virus exposure in humans suggests that 2.3–13.7% of workers exposed to poultry in China have detectable antibodies against the H9 influenza hemagglutinin protein^9^.

The ongoing evolution of H9N2 influenza viruses in nature poses a significant public health risk. Many avian H9N2 influenza viruses can replicate in mammals without prior adaption^10^,^11^,^12^. Moreover, the internal genes of an H5N1 virus isolated from a human in Hong Kong in 1997 may have originated from H9N2 influenza viruses^13^,^14^. Similarly, the H7N9 avian influenza viruses isolated from humans in China in 2013 carried six internal genes from avian H9N2 viruses^15^. While H9N2 AIVs have not yet acquired the capacity for human-to-human transmission, major questions remain concerning the capacity of these viruses to replicate in and transmit among mammals.

Several experimental approaches have been used to explore the capacity of avian influenza subtypes to acquire a transmissible phenotype. In general, these approaches rely upon the genetic reassortment of an avian subtype virus with a human influenza virus, the adaptation of an avian subtype virus in mammals through serial passage, or a combination of both of the above. These approaches have been used to demonstrate that reassortant H5N1 viruses containing 2009/H1N1 genes were capable of respiratory droplet-mediated transmission in guinea pigs and ferrets^16^,^17^,^18^. Similarly, reassortant H9N2 viruses containing genes from a human H3N2 or 2009/H1N1 virus were transmissible in ferrets^19^,^20^, and serial passage of an H7N1 influenza virus resulted in adaptive mutations enabling airborne transmission in ferrets^21^.

The ability of avian influenza viruses to transmit between mammalian hosts appears to be require multiple viral features, including α2,6-linked sialic acid (SA) receptor binding (the human-type receptor) as well as high levels of heat stability and increased polymerase activity in human cells^16^,^17^,^18^,^21^,^22^,^23^,^24^,^25^,^26^,^27^,^28^. H9N2 influenza viruses bearing a threonine at amino acid position 155 or leucine at amino acid position 226 (H3 numbering) of the HA protein possess the ability to predominantly bind α2,6-linked SA moieties^29^,^30^,^31^ and are important for transmissibility of H9N2 virus in a ferret model^10^. Although more than 80% of H9N2 influenza virus isolates from China possess a leucine at HA position 226 (http://blast.ncbi.nlm.nih.gov/Blast.cgi), the limited number of reported human H9N2 infections suggests that α2,6-linked SA receptor binding is not sufficient for infection or human-to-human transmission.

Serial passage of AIVs in mammalian hosts can result in adaptive viral changes that confer an increased replication capacity in mammalian cells and may enhance transmission between hosts. We sought to explore the impact of mammalian adaptation on transmission dynamics of an H9N2 AIV using a guinea pig model. A wild-type H9N2 AIV isolate with affinity for both α2,6-linked SA (the human-type receptor) and α2,3 -linked SA (the avian-type receptor) moieties was serially passaged through guinea pigs. After every third passage, we evaluated the ability of the adapted virus to transmit by direct contact in guinea pigs. Guinea pig-adapted H9N2 viruses acquired the ability to transmit by direct contact, but were less efficiently transmitted via respiratory droplets in guinea pigs. These findings suggest that mammalian adaptation of H9N2 viruses may generate viruses capable of transmission by direct contact in mammals, although additional amino acid substitutions are likely required for efficient airborne transmission.

## Results

### Virulence and receptor binding specificity of wild-type H9N2 AIVs

We evaluated three H9N2 AIVs, A/Chicken/Shandong/Li-2/2010 (H9N2) (abbreviated as SD2/H9N2), A/Chicken/Shandong/Li-3/2010 (H9N2) (abbreviated as SD3/H9N2), and A/Chicken/Jilin/Hu-3/2006 (H9N2) (abbreviated as JL3/H9N2), isolated from chickens for properties of virulence in mice and receptor binding specificity. Each of the three H9N2 viruses displayed MLD_50_ values >6.5 (expressed as log_10_EID_50_, Table 1). The receptor binding specificity of the three viruses was preliminarily determined by evaluating the ability of each H9N2 virus to agglutinate untreated and α2,3-sialidase-treated chicken red blood cells (CRBCs) as previously described^18^. As expected, each avian-origin H9N2 virus could agglutinate untreated CRBCs (Table 1). Whereas the JL3/H9N2 virus could not agglutinate α2,3-sialidase-treated CRBCs, the SD2/H9N2 and SD3/H9N2 viruses could agglutinate α2,3-sialidase-treated CRBCs, suggesting these viruses could bind the remaining α2,6-linked sialic acid moieties (Table 1).

**Figure 1:**
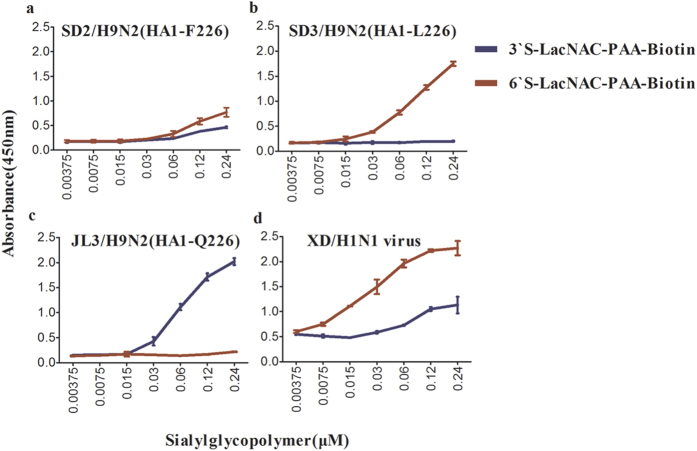
Characterization of the receptor binding specificity of wild-type H9N2 viruses and XD/H1N1 virus. Each indicated virus was assessed for binding to an α2,6-linked SA (red lines) and α2,3-linked SA (blue lines) glyocpolymer in a solid phase binding assay. Viruses used include SD2/H9N2 (**a**), SD3/H9N2 (**b**), JL3/H9N2 (**c**), and XD/H1N1 (**d**). The amino acid residue at HA1 position 226 is indicated for each virus.

A solid-phase binding assay was used to further assess the receptor binding specificities of each virus. The wild-type SD2/H9N2 virus, possessing a phenylalanine residue at HA position 226, preferentially recognized the human-type receptor and only weakly recognized the avian-type receptor (Fig. 1a). The SD3/H9N2 virus, possessing a leucine residue at HA position 226, bound only to the human-type receptor (Fig. 1b). The JL3/H9N2 virus, possessing a glutamine at HA position 226, bound only to the avian-type receptor (Fig. 1c). The A/Changchun/01/2009 (H1N1) virus, abbreviated as XD/H1N1, served as a control virus and displayed preferential binding to the human-type receptor (Fig. 1d). These results are consistent with previous studies that demonstrated that H9N2 viruses carrying HA-L226 preferentially recognize human-type receptors, while viruses bearing HA-Q226 preferentially recognize avian-type receptors^29^,^30^.

### Wild-type H9N2 AIVs do not transmit by direct contact in guinea pigs

**Figure 2:**
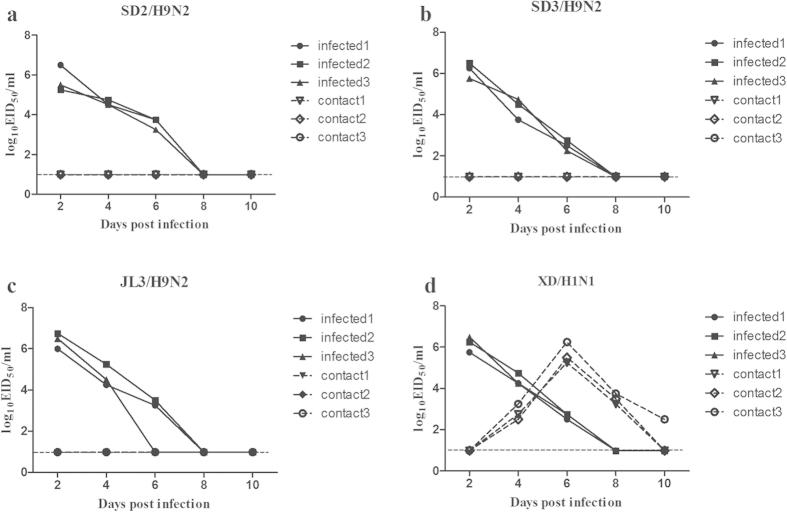
Assessment of direct contact-mediated transmissibility of wild-type H9N2 AIVs in guinea pigs. Groups of three guinea pigs were inoculated i.n. with 10^6^ EID_50_ SD2/H9N2 (**a**), SD3/H9N2 (**b**), JL3/H9N2 (**c**), or the human influenza virus XD/H1N1 (**d**). Twenty-four hours later, a naïve contact guinea pig was placed in each cage with an inoculated animal. Nasal washes were collected every two days from all animals beginning 2 days post-infection to detect the presence of virus. Each line represents the virus titer for an individual animal. The dashed lines indicate the lower limit of detection.

We next evaluated the capacity of each H9N2 virus to transmit between guinea pigs in a direct contact model. Groups of three guinea pigs were inoculated i.n. with 10^6^ EID_50_ of SD2/H9N2, SD3/H9N2, or JL3/H9N2. The XD/H1N1 virus was used as a positive control for viral transmission between guinea pigs. Each inoculated guinea pig was housed in a separate cage and isolator. Twenty-four hours after inoculation, a naïve guinea pig was placed into each cage with an inoculated animal. To assess productive transmission, we evaluated nasal washes collected from the naïve contact guinea pigs at various time points post-contact for the presence of virus and assessed serum for HI antibody responses after 21 days. While virus was detected in the nasal washes of all inoculated guinea pigs following i.n. inoculation with each H9N2 virus from days 2 to 4 post-infection, none of the naive contact guinea pigs seroconverted or had detectable virus in nasal washes (Fig. 2a–c and Table 1). In contrast, the XD/H1N1 virus was recovered from the nasal washes of 3/3 contact animals beginning on day 4 post-infection, indicating productive transmission via direct contact (Fig. 2d). These results show that the three H9N2 viruses were not capable of efficient transmission by direct contact in guinea pigs despite differences in HA binding preferences.

### Adapted H9N2 viruses transmit between guinea pigs by direct contact

We next sought to determine whether a transmissible virus could be generated through experimental adaptation of H9N2 AIVs via serial passage in guinea pigs. Novel avian-origin H7N9 viruses isolated from humans in China in 2013 possessed the capacity to recognize both human- and avian-type influenza receptors^32^,^33^. We therefore focused subsequent experiments on the SD2/H9N2 virus which could recognize both human- and avian-like receptors to explore the impact of mammalian adaptation on transmission efficiency.

The SD2/H9N2 virus was serially passaged for fifteen rounds in guinea pigs. The ability of the adapted viral population to transmit in a guinea pig direct contact model was assessed after every third passage. Adapted SD2/H9N2 viruses generated after passage 9 (P9), passage 12 (P12) and passage 15 (P15) acquired the ability to transmit to naive guinea pigs in a direct contact model (Fig. S1). The P9 and P12 virus was transmitted from infected guinea pigs to two of three paired naive contact animals (Fig. S1a-b). In each instance, the virus was first detected in contact animals on day 4 post-infection (Fig. S1a-b). By passage 15, virus was transmitted to all three contact animals (Fig. S1c). Virus was detected in the nasal wash collected from one contact animal on day 2 post-infection and in the other two contacts by day 4 post-infection. Peak virus titers were recovered from all three contact animals on day 6. These data demonstrate that serial passage of an avian-origin H9N2 virus in guinea pigs results in adaptive changes resulting in the acquisition of a transmissible phenotype in a guinea pig direct contact model.

### Assessment of amino acid substitution combinations enabling H9N2 transmission by direct contact in guinea pigs

We sequenced the complete genome of the viral population recovered in nasal washes of inoculated guinea pigs after every third passage to identify amino acid substitutions (Table 2). After the 15^th^ passage, five amino acid substitutions were identified, including PB2-D195N, HA1-Q227P, HA2-D46E, NP-E434K, and M1-Q211R (Table 2). These amino acid substitutions correlated with productive H9N2 viral transmission by direct contact in 3/3 guinea pig pairs (Fig. S1c). Of these five substitutions, only PB2-D195N, HA1-Q227P, and NP-E434K substitutions were present after passage 9, in which we observed transmission between guinea pigs in 2/3 pairs. When compared to the P9 virus, the P6 virus differed only in that the PB2-D195N substitution was detectable but had not yet become dominant, although the P6 virus did not transmit between any of the three guinea pig pairs. Notably, a second independent serial passage of SD/H9N2 virus in guinea pigs yielded an adapted H9N2 virus at passage 15 that possessed an identical constellation of amino acid substitutions as described above and was similarly transmitted between 3/3 guinea pig pairs by direct contact (data not shown). These data suggest that PB2-D195N, HA1-Q227P, and NP-E434K are sufficient to confer a transmissible phenotype to the SD2/H9N2 virus although the HA2-D46E and M1-Q211R substitutions may enhance transmission efficiency.

**Figure 3:**
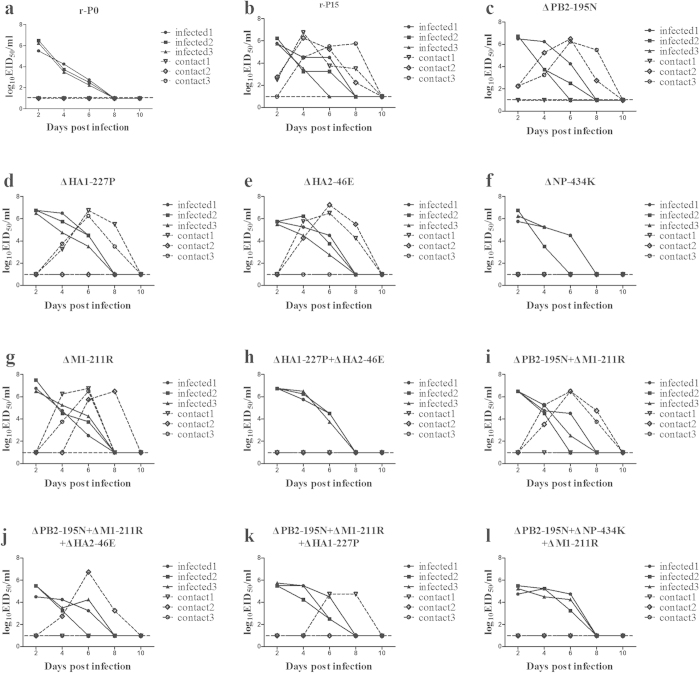
Assessment of amino acid substitution combinations on direct contact transmission in guinea pigs. Recombinant viruses harboring different combinations of amino acid substitutions identified in the guinea-pig adapted H9N2 virus were generated and assessed for their ability to transmit via direct contact in guinea pigs. (**a**) Contact transmission of rescued SD2/H9N2 virus; (**b**) Contact transmission of recombinant virus bearing all five amino acid substitutions identified in the P15 virus (PB2-D195N, HA1-Q227P, HA2-D46E, NP-E434K, and M1-Q211R) in the backbone of SD2/H9N2; (**c**–**l**) Contact transmission of recombinant versions of the r-P15 adapted viruses lacking one or more of the amino acid substitutions found in the P15 virus. Omitted amino acid substitutions are indicated by a Δ and the specific substitution.

We next sought to identify the contribution of different amino acid substitutions on SD2/H9N2 transmission efficiency using reverse genetics. As expected, a recombinant virus containing all five of the identified amino acid substitutions (named r-P15) was productively transmitted from inoculated guinea pigs to 3/3 paired naive contact animals (Fig. 3b). Notably, the two contact guinea pigs shed SD2/H9N2 virus in nasal wash beginning 1 day post-contact, suggesting early and efficient transmission.

We next generated a panel of recombinant viruses in which one or more of the five adaptive substitutions identified in the P15 virus was omitted and evaluated transmission efficiency in the guinea pig contact model. Recombinant viruses lacking either HA1-Q227P, HA2-D46E or PB2-D195N replicated well in inoculated guinea pigs, but productively transmitted to only 2/3 naive contacts by direct contact in each case, suggesting that these substitutions are not strictly required for transmission, but may enhance transmission efficiency (Fig. 3c–e). A recombinant virus lacking the NP-E434K substitution replicated well in inoculated guinea pigs, but lost the ability to transmit to naive contact animals (Fig. 3f). A recombinant virus lacking the M1-Q211R substitution was productively transmitted to 3/3 naive contacts, demonstrating that this adaptive change was not required for transmission (Fig. 3g).

The above results suggest that NP-E434K and either HA1-Q227P or HA2-D46E substitutions are required for contact transmission of the adapted virus in guinea pigs. To test the impact of these amino acid substitutions in combination, we generated recombinant viruses harboring NP-E434K, HA1-Q227P, and HA2-D46E by reverse genetics. The HA1-Q227P-, HA2-D46E- and NP-E434K-containing recombinant virus was transmitted in 2/3 guinea pig pairs in the direct contact model (Fig. 3i). In contrast, recombinant viruses bearing NP-E434K and either HA1-Q227P or HA2-D46E showed reduced transmission frequency, being transmitted in 1/3 guinea pig pairs (Fig. 3j,k). These results demonstrate that NP-E434K and either HA1-Q227P or HA2-D46E are sufficient for direct contact transmission in guinea pigs.

### HA1-Q227P and HA2-D46E substitutions alter receptor binding specificity

**Figure 4:**
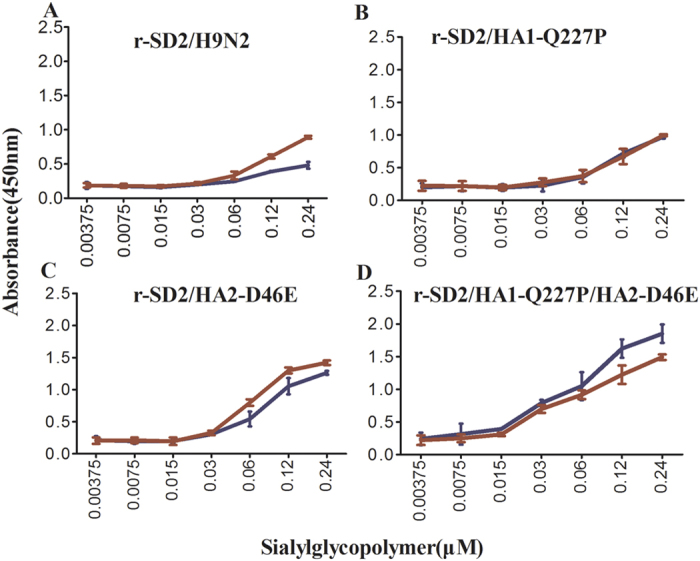
Characterization of the receptor binding specificity of recombinant H9N2 AIVs possessing HA amino acid substitutions identified in adapted viruses. Recombinant viruses incorporating the indicated amino acid substitution(s) in the background of SD2/H9N2 were generated by reverse genetics and assessed for binding to an α2,6-linked SA (red lines) and α2,3-linked SA (blue lines) glyocpolymer in a solid phase binding assay. Recombinant viruses used include r-SD2 (**a**), r-SD2/HA1-Q227P (**b**), r-SD2/HA2-D46E (**c**), and r-SD2/HA1-Q227P/HA2-D46E (**d**).

The impact of HA1-Q227P and HA2-D46E substitutions on H9N2 transmission efficiency in guinea pigs prompted an evaluation of these substitutions on HA receptor binding specificity. HA1-Q227P is located immediately adjacent to an amino acid previously recognized to play a role in receptor preference (amino acid position 226; H3 numbering), whereas HA2-D46E is located in the α-helix of the HA2 subunit. Solid-phase binding assays were performed to determine whether these substitutions affected virus receptor preference. The HA1-Q227P and HA2-D46E substitutions independently increased the ability of each virus to bind an avian-type α2,3 SA receptor when compared to the parental r-SD2/H9N2 virus (Fig. 4a–c). The combination of two substitutions (HA-Q227_1_P/D46_2_E) displayed enhanced binding to both avian- and human-type receptors (Fig. 4d). These results show that HA1-Q227P substitution increased the ability of the adapted virus to bind to the avian-type receptor, while the HA2-D46E substitution enhanced binding to both avian- and human-type receptors.

### HA2-D46E increases viral thermostability

**Figure 5:**
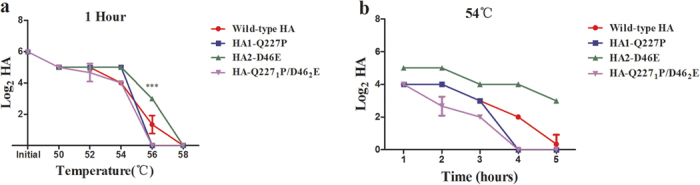
Impact of adaptive amino acid substitutions on H9N2 thermostability. Sixty-four HA units of each indicated virus were incubated at different temperatures for 1 hour (**a**) or at 54 °C for 1–5 hours (**b**) and assessed for hemagglutination activity. Results are means ± S.D. from three independent experiments. (***P < 0.001; one-way ANOVA test).

We evaluated the thermostability of viruses with wild-type HA or variant HA proteins harboring the HA1-Q227P and/or HA2-D46E substitutions as previously described^22^,^34^. Retention of virus-mediated haemagglutination activity following heat-treatment is a measure of HA thermostability. The parental r-SD2/H9N2 virus and viruses containing HA1-Q227P and/or HA2-D46E substitutions were incubated at varied temperature (50 °C–58 °C) for one hour (Fig. 5a) or at 54 °C for 1–5 hours (Fig. 5b). Introduction of the HA1-Q227P substitution slightly decreased the thermostablity of HA as compared to the parental r-SD2/H9N2 virus (Fig. 5a,b). In contrast, a virus bearing the HA2-D46E substitution retained more haemagglutination activity than the parental virus following one hour incubation at 56 °C (Fig. 5a). Additionally, the HA2-D46E virus lost haemagglutination activity more slowly than the parental virus at 54 °C (Fig. 5b). A virus harboring both HA1-Q227P and HA2-D46E substitutions lost haemagglutination activity faster when compared to the parent virus at 54 °C (Fig. 5b). These results show that a HA2-D46E substitution had a stabilizing effect on the HA of the adapted virus.

### PB2-D195N and NP-E434K mutations independently enhance viral RNA polymerase activity *in vitro*

**Figure 6:**
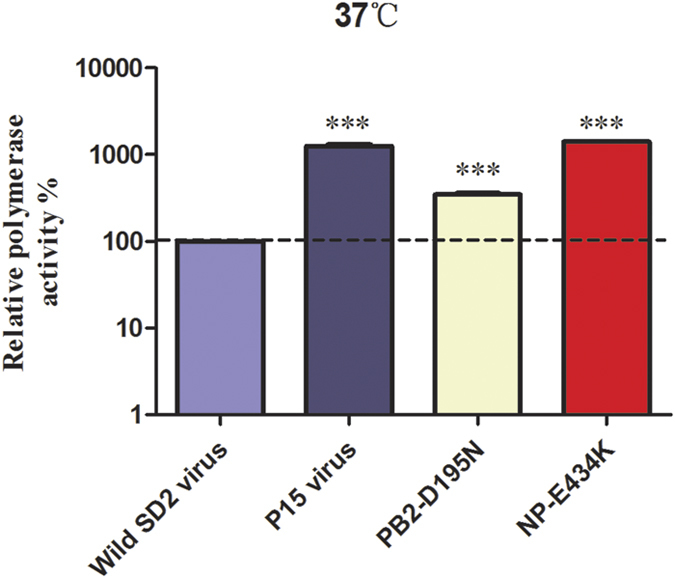
PB2-D195N and NP-E434K independently enhance polymerase activity *in vitro*. 293T cells were transfected with PB2-, PB1-, PA- and NP-encoding plasmids of wild-type H9N2, the P15 virus, or wild-type H9N2 with the indicated amino acid substitutions together with the p-Luci luciferase reporter plasmid and the Renilla internal control plasmid. Values shown are the mean ± standard deviation of three independent experiments and are standardized to that of wild-type H9N2 virus (100%, indicated by the dashed line). ***P < 0.001; one-way ANOVA test.

We next assessed whether adaptation in guinea pigs led to enhanced viral RNA polymerase activity. The polymerase activity of the parental SD2/H9N2 virus was compared to that of the P15 adapted virus using a mini-replicon assay in 293T cells at 37 °C (Fig. 6). The polymerase activity of the P15 virus was 12.5-fold higher than that of the wild-type virus (*P* < 0.001; one-way ANOVA test). Since the P15 virus possessed two amino acid substitutions in the viral replication complex, PB2-D195N and NP-E434K, we respectively assessed the impact of each single substitution on viral polymerase activity. The PB2-D195N substitution increased polymerase activity by about 3.5-fold (*P* < 0.001; one-way ANOVA test), while the NP-E434K substitution resulted in 14-fold higher polymerase activity when compared to wild-type polymerase activity (*P* < 0.001; one-way ANOVA test). These results show that the PB2-D195N and NP-E434K substitutions, which were required for direct contact transmission, increased viral polymerase activity.

### Adapted H9N2 AIV inefficiently transmits by respiratory droplets in guinea pigs

**Figure 7:**
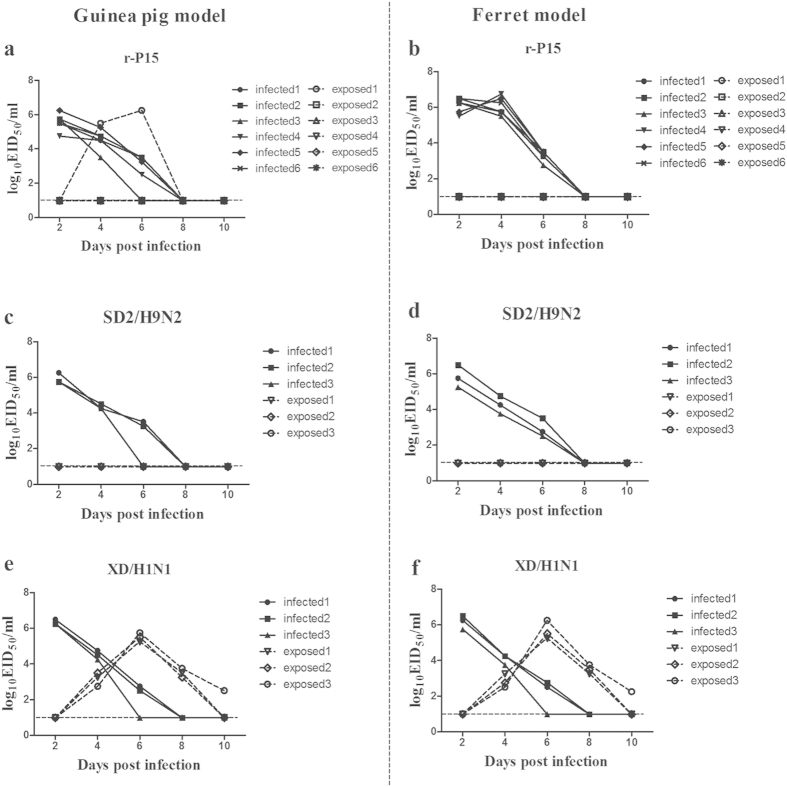
Assessment of droplet-mediated transmission of r-P15 virus in guinea pigs and ferrets. Guinea pigs or ferrets were inoculated i.n. with 10^6^ EID_50_ of r-P15 virus (**a**,**b**, n = 6), SD2/H9N2 virus (**c**,**d**, n = 3), or XD/H1N1 virus (n = 3). Twenty-four hours later, a naïve contact animal was placed in a cage adjacent to each inoculated animal. Nasal washes were collected every two days from all animals beginning 2 days post-infection for detection of virus shedding. Each line represents the virus titer from an individual animal. The dashed lines indicate the lower limit of detection.

We next assessed whether the adapted H9N2 virus was capable of transmission via respiratory droplets in both a guinea pig and ferret model. Guinea pigs or ferrets were intranasally inoculated with 10^6^ EID_50_ of the r-P15 virus, or the wild-type SD2 virus or XD/H1N1 virus as transmission controls. Twenty-four hours later, naïve contact animals were placed in cages next to the inoculated animals. In the guinea pig model, all inoculated animals had detectable virus in nasal washes on days 2 and 4 post-infection (Fig. 7a). Virus was transmitted between one of six guinea pig pairs, as indicated by the presence of virus in the nasal wash of one contact guinea pig on days 4 and 6 post-infection (Fig. 7a). No additional mutations were found in the virus isolated from the exposed guinea pig when compared with the inoculated virus (r-P15). In the ferret model, all inoculated animals had detectable virus in nasal washes on days 2, 4, and 6 post-infection, although there was no evidence of viral transmission between ferrets, as indicated by a lack of detectable virus in nasal washes (Fig. 7b). Throughout the experimental period, intranasally inoculated ferrets did not display elevated body temperatures or any overt clinical signs (data not shown). With the exception of the one guinea pig in which respiratory droplet-mediated H9N2 transmission occurred, no contact animals seroconverted following exposure to inoculated animals. As expected, the wild-type SD2/H9N2 virus did not transmit to naïve contact guinea pigs or ferrets by respiratory droplets and contact animals did not show evidence of seroconversion (Fig. 7c,d). In contrast, the XD/H1N1 control virus was detected in the nasal wash of all three contact guinea pigs and ferrets from days 4 to 8 post-infection, indicating productive viral transmission between guinea pigs and ferrets via respiratory droplets (Fig. 7e,f). These results suggest that the adapted H9N2 virus is less efficiently transmitted by respiratory droplets in guinea pigs and ferrets as compared to transmission by direct contact.

### The pathogenicity and antigenicity of adapted H9N2 virus

To explore whether the pathogenicity of the adapted H9N2 virus changed, groups of three 5 to 7-week old BALB/c mice were inoculated i.n. with 10^6^ EID_50_ of wild-type virus or the r-P15 virus. The wild-type virus did not elicit weight loss in mice, whereas mice infected with the r-P15 virus displayed modest weight loss that did not exceed 10% of the initial body weight (data not shown). These results suggest that the adaptive changes acquired during serial passage of the H9N2 virus in guinea pigs did not substantially enhance viral virulence in mice.

We evaluated changes in viral antigenicity that may have occurred during serial passage of the H9N2 virus in guinea pigs as previously described^35^. No significant antigenic differences were found when the wild-type virus was compared to the P9 virus generated after nine serial passages in guinea pigs (R = 0.71). A modest change in antigenicity was noted when the wild-type virus was compared to the P15 virus generated after 15 passages in guinea pigs (R = 0.63). These results showed the adapted H9N2 virus in guinea pig did not cause substantial antigenic changes.

## Discussion

Previous studies have demonstrated that amino acid substitutions acquired during mammalian adaptation of AIVs can enhance transmission efficiencies in mammals^17^,^21^. Here, we have adapted an avian H9N2 virus that initially displayed an ability to recognize both α2,3 and α2,6-linked SA residues via serial passage in guinea pigs. Three amino acid substitutions introduced into the H9N2 AIV during serial passage (HA1-Q227P, HA2-D46E, and NP-E434K or HA1-Q227P, NP-E434K, and PB2-D195N) were sufficient to increase contact transmission efficiency between guinea pigs (2/3 pairs). Importantly, viruses with the NP-E434K substitution and either HA1-Q227P or HA2-D46E were still transmitted by direct contact, although transmission only occurred in only 1/3 pairs in each instance (Fig. 3j,k).

We explored the mechanistic basis by which these three amino acid substitutions may contribute to the ability of the adapted H9N2 virus to transmit by direct contact. Receptor binding preference is widely recognized to be an important factor for influenza virus transmission in different animal model systems^18^,^19^,^23^,^24^,^36^. However, a highly pathogenic strain of H7N1 avian influenza can be adapted to become airborne transmissible in mammals without mutations altering the receptor specificity^21^. We found that the HA1-Q227P and HA2-D46E substitutions independently increased the ability of each virus to bind an avian-type α2,3 SA receptor when compared to the parental SD2/H9N2 virus with minimal impact on recognition of a human-type α2,6 SA receptor (Fig. 5). Increases in receptor binding ability to avian-type receptors may be critical for transmission in the guinea pig model, as both avian-type and human-type receptors are widely distributed throughout the upper respiratory tract of guinea pigs^36^,^37^. Importantly, the HA2-D46E substitution was shown to increase virus binding to both 2,3 and 2,6-linked SA residues, indicating that H9N2 receptor binding specificity can be affected by amino acid substitutions at locations other than those associated with the receptor binding pocket. Further studies will be required to assess the impact of these amino acid substitutions on contact-mediated transmission of H9N2 AIV in mammalian models in which receptor expression in the upper respiratory tract more closely resembles that in humans.

In addition to receptor binding preference, HA stability and polymerase activity have also been previously identified as factors that impinge upon the transmission capacity of influenza viruses. The HA2-D46E substitution enhanced viral thermostability, as shown by the ability of viruses harboring the HA2-D46E substitution to retain hemagglutination activity following heat treatment when compared to the wild-type virus (Fig. 5). Although HA2-D46E substitution alone increased HA stability when compared to the wild type virus, the P15 virus bearing both HA1-Q227P and HA2-D46E substitution in HA was less stable compared to the wild type virus. This result suggests that HA2-D46E may be a compensatory mutation for Q227P in HA. The viral ribonucleoprotein (vRNP) plays an important role in adaption of influenza virus in hosts. PB2-627K is well-recognized marker associated with enhanced viral replication in mammalian cells and transmission^25^,^36^,^38^. The adapted virus generated after 15 passages in guinea pigs possessed two amino acid substitutions in proteins that compose the vRNP complex, NP-E434K and PB2-D195N. The NP-E434K substitution was absolutely required for direct contact transmission between guinea pigs, whereas a recombinant version of the adapted H9N2 virus lacking PB2-D195N displayed reduced transmission efficiency and transmitted between 2/3 guinea pig pairs. Using an *in vitro* assay to characterize viral polymerase activity, we showed that the PB2-D195N and NP-E434K substitution independently enhanced polymerase activity in mammalian cells. However, the combination of these two substitutions did not further increase the viral polymerase activity compared to the effect of single NP-E434K substitution, implying the role of PB2-D195N substitution should be further studied (Fig. 6). Notably, searching influenza sequences deposited in GenBank revealed that the NP-E434K substitution has been identified in a natural H9N2 AIV isolate and the PB2-195N residue is present in avian H5N1 viruses. Though the NP-E434K substitution enhanced *in vitro* polymerase activity, it is unclear how this translates to enhanced viral transmission. Changes in replication efficiency may contribute to higher overall viral titers in inoculated animals, influence viral replication at different sites within the respiratory tract, and impact virus shedding. Alternatively, increased polymerase activity may allow viruses to more efficiently establish infection during transmission.

Collectively, these results show that three amino acid substitutions are sufficient to enable direct contact transmission of an H9N2 AIV in guinea pigs and provide mechanistic insights into the viral attributes that may be required for contact transmission. While the adapted H9N2 virus generated after 15 serial passages in guinea pigs was efficiently transmitted by direct contact, we found evidence of transmission by respiratory droplets in only 1/6 guinea pig pairs and in 0/6 ferret pairs. This suggests that additional molecular features are required for respiratory droplet-mediated transmission of H9N2 AIVs.

Recently, novel H7N9 and H10N8 viruses isolated from humans in China were shown to possess genes derived from H9N2 AIVs, suggesting that H9N2 AIVs in poultry can contribute gene segments during reassortment events leading to the generation of novel avian influenza virus that infect humans^15^,^39^. Poultry markets may serve as an environment supporting genetic reassortment of influenza viruses and accelerate the generation of novel influenza viruses^40^,^41^. Additionally, the high prevalence of H9N2 AIVs in poultry provides opportunities for sporadic transmission to humans. Our results highlight that mammalian adaptation of H9N2 AIVs generates amino acid substitutions that enable transmission by direct contact in animal models, although respiratory droplet-mediated transmission may require additional substitutions that are not elicited by adaptation via serial passage. These findings further underscore the pandemic potential of H9N2 avian influenza viruses and provide mechanistic insights into the transmissibility of influenza virus among mammalian hosts. The amino acid substitutions associated with contact transmission in this study may inform surveillance efforts to detect the emergence of H9N2 isolates with an increased propensity for mammalian transmission.

## Materials and Methods

### Ethics statement

All animal studies were approved by the Review Board of Military Veterinary Research Institute of the Academy of Military Medical Sciences (Number SYXK2009-045). The protocol of the study was conducted in accordance with guidelines of animal welfare of World Organization for Animal Health.

### Facilities

Studies with H9N2 virus and adapted variants were performed in a biosecurity level 3 laboratory approved by the Military Veterinary Research Institute of the Academy of Military Medical Sciences. The research program, procedures, occupational health plan, security and facilities are reviewed by the Academy of Military Medical Sciences.

### Viruses

All H9N2 viruses used in the study were isolated from chickens and include: A/Chicken/Shandong/Li-2/2010(H9N2), A/Chicken/Shandong/Li-3/2010 (H9N2), and A/Chicken/Jilin/Hu-3/2006(H9N2). The 2009 pandemic H1N1 influenza virus, A/Changchun/01/2009(H1N1), was acquired from the Academy of Military Medical Sciences. Viruses were stored at −80 °C.

### Animal studies

Hartley strain female guinea pigs weighing 300–350 g (Vital River Laboratories, Beijing, China) and 4-month-old male ferrets (Wuxi Cay Ferret Farm, China) serologically negative for prior influenza virus infection were used in these studies. Ketamine (20 mg/kg) and xylazine (1 mg/kg) were used to anesthetize animals by intramuscular injection.

To evaluate the efficiency of each virus to transmit by direct contact, groups of three guinea pigs were inoculated intranasally (i.n.) with 10^6^ EID_50_ of SD2/H9N2, SD3/H9N2, JL3/H9N2 or XD/H1N1 in 300 μl. Each inoculated guinea pig was housed in a separate cage in an independent isolator. Twenty-four hours later, one naive guinea pig was introduced into each cage with an experimentally inoculated animal. Nasal washes were collected from each guinea pig at two day intervals, beginning two days post-inoculation (one day post-contact for naïve contact animals). Virus titers were determined by titration in eggs. To prevent inadvertent physical transmission of virus by investigators, the contact guinea pigs were handled first, and all materials used to handle and manipulate the guinea pigs during nasal wash collection were changed between animals. The ambient conditions for these studies were set at 20–22 °C with 30–40% relative humidity. Horizontal airflow in the isolator was set a speed of 0.1 m/s. Sera were collected from all animals after 21 days for hemagglutinin inhibition (HI) antibody detection.

To measure the capacity of each virus to transmit by respiratory droplets, groups of six guinea pigs or ferrets were inoculated i.n. with 10^6^ EID_50_ of SD2/H9N2 virus or a guinea pig-adapted virus. As controls, groups of three guinea pigs or ferrets were inoculated i.n. with 10^6^ EID_50_ of SD2/H9N2 virus or XD/H1N1 virus. Each inoculated animal was housed in a separate cage in an independent isolator. After 24 hours, a naïve animal was placed in a chamber adjacent to the chamber housing the inoculated animal (separated by 2cm). Nasal washes were collected at 2 day intervals, beginning on day 2 post-inoculation (1 day post-contact). Viral titers were determined by titration in eggs. Sera were collected from all animals on day 21 for HI antibody detection.

To evaluate the pathogenicity of H9N2 AIVs in mice, groups of three 5 to 7 week-old female BALB/c mice (Vital River Laboratories, Beijing, China) were lightly anesthetized with dry ice and inoculated i.n. with 10^6^ EID_50_ of SD2/H9N2, SD3/H9N2, JL3/H9N2, or guinea pigs adapted virus. Mice were monitored daily for weight loss and mortality for 14 days. Mice that lost more than 25% of the initial body weight were recognized as experimental end point and humanely euthanized. Serum was collected from each mouse on day 21 for HI antibody detection.

### Serial passage of SD2/H9N2 virus in guinea pigs

SD2/H9N2 was serially passaged in two independent lines of guinea pigs in order to identify amino acid substitutions associated with mammalian adaptation of an avian-origin H9N2 virus. In brief, the first guinea pig in each independent line was inoculated i.n. with 300 μl containing 10^6^ EID_50_ of SD2/H9N2 virus. Three days later, a naïve guinea pig was inoculated i.n. with 300 μl of nasal wash from the previously inoculated guinea pig. Fifteen successive passages were performed. Successful infection of each guinea pig in the series was confirmed by titrating virus present in the nasal washes from 2 to 5 days p.i. in eggs. As the constellation of adaptive amino acid substitutions in viruses generated each independent line of guinea pigs was found to be identical after 15 passages, one lineage of adapted H9N2 was used for subsequent analyses. For the selected adapted lineage, the viral genome was sequenced after every third passage and the capacity for transmission assessed in the guinea pig contact model as described above.

### Sequence analysis

Viral RNA was extracted from the nasal wash of guinea pigs on day 3 post-inoculation after every third passage. Viral genes were amplified by reverse transcription-PCR using QIAGEN One-Step RT-PCR Kit (Qiagen, Germany) according to the manufacturers’ instructions. Amplified cDNAs were sequenced by Comate Bioscience Corporation.

### Virus rescue and site-directed mutagenesis

Each of the eight SD2/H9N2 viral gene segments was amplified and rescued by using the pBD plasmid as previously described^42^. Mutations encoding specific amino acid substitutions were introduced using site-directed mutagenesis (Invitrogen, USA). All the constructs were verified by sequencing. The whole sequences of the wild-type SD2/H9N2 virus, HA gene of SD3/H9N2 virus and HA gene of JL3/H9N2 virus were deposited in GenBank under accession numbers KM411625-KM411634.

### Preparation of sialidase-treated CRBCs

Sialidase-treated Chicken red blood cells (CRBCs) were prepared as previously described^18^ with modified. CRBCs were washed and diluted to 10% (vol/vol) in PBS. 0.1 ml CRBCs were incubated with 10 μl (50 U/ml) of α2,3 –sialidase (Takara, Japan) for 15 min at 37 °C, washed three times in PBS, and re-suspended in PBS.

### Solid-phase binding assay

Virus receptor specificity was evaluated using a direct solid phase assay as described previously^25^,^36^. Different concentrations of two biotinylated glycans (Neu5Acα2-3Galβ1-4GLcNAcβ1-4GlcNAcβ-PAA-biotin and Neu5Acα2-6Galβ1-4GLcNAcβ1-4GlcNAcβ-PAA-biotin; GlycoTech Corporation, USA) were plated in wells of a streptavidin-coated 96-well plate (Pierce, USA) and incubated at 4 °C overnight. 64 HA units of influenza virus in PBS was added after washing and incubated at 4 °C overnight. A chicken polyclonal antiserum against H9N2 or 2009/H1N1 were added and incubated for 4 h at 4 °C. HRP-linked rabbit-anti-chicken antibody (Sigma-Aldrich, USA) was added for 2 h at 4 °C. TMB substrate (Sigma-Aldrich, USA) was used and the optical density at 450 nm was recorded.

### Thermostability assay of HA

The thermostability of wild-type virus and viruses harboring amino acid substitutions in the HA protein was evaluated as follows. Sixty-four HA units of each virus were incubated at 50 °C–58 °C in 2 degree increments for 60 minutes, or were incubated at 54 °C for 1–5 hours. Heat-treated viruses were evaluated by using a haemagglutination assay.

### Polymerase activity assay

A dual-luciferase reporter assay system (Promega, USA) was used to compare the polymerase activities of viral RNP complexes. The PB2, PB1, PA and NP gene segments of wild-type and the adapted virus generated following 15 passages in guinea pigs were cloned into the pCDNA3.1 expression plasmid and transfected to 293T cells with the p-Luci luciferase reporter plasmid and the Renilla internal control plasmid. Cell lysates were analyzed 36 hours after transfection to measure firefly and Renilla luciferase activities. Values shown are the mean ± standard deviation (SD) of three independent experiments and are normalized to the values obtained for wild-type virus at 37 °C (set as 100%).

### Antibody detection and antigenicity analysis

Sera were treated with *Vibrio cholerae* (Denka-Seiken, Japan) receptor-destroying enzyme before hemagglutination inhibition (HI) tests. For antigenicity analysis, the sera were collected from BALB/c mice inoculated i.n. with 10^6^ EID_50_ of wild virus, P9 virus and P15 virus on day 21 p.i. The antigenic relatedness between the different viruses was expressed as R-value based on the Archetti and Horsfall formula^35^,^43^.

### Statistical analyses

Statistically significant differences between experimental groups were evaluated using analysis of variance (one-way ANOVA, Dunnett) with the GraphPad Prism 5 software package (GraphPad Software Inc., La Jolla, CA, USA). *P*-values less than 0.05 were considered statistically significant.

## Additional Information

**How to cite this article**: Sang, X. *et al.* Adaptation of H9N2 AIV in guinea pigs enables efficient transmission by direct contact and inefficient transmission by respiratory droplets. *Sci. Rep.* **5**, 15928; doi: 10.1038/srep15928 (2015).

## Supplementary Material

Supplementary Information

## Figures and Tables

**Table 1:** Pathogenicity, receptor binding preference and transmissibility of wild-type H9N2 AIVs.

Virus (aberration)	MLD_50_^a^	Receptor binding specificity^b^		HA 226 residue^c^	Transmission^d^
		0.5% CRBCs	1% Siaα2,6-CRBC		
A/Chicken/Shandong/Li-2/2010(SD2/H9N2)	>6.5	64	32	F	0/3
A/Chicken/Shandong/Li-2/2010 (SD3/H9N2)	>6.5	64	32	L	0/3
A/Chicken/Jilin/Hu-3/2006(JL3/H9N2)	>6.5	64	<2	Q	0/3

^a^Pathogenicity for mice was evaluated as MLD_50_ value (expressed as log_10_EID_50_).

^b^Hemagglutination test of viruses for chicken red blood cells (CRBCs) possessing both α2,3-linked sialic acids (SAs) and α2,6-linked SAs, or CRBCs treated with α2,3-Sialidase, leaving predominantly α2,6-linked SAs.

^c^The amino acid residues at HA amino acid position 226 (H3 HA numbering).

^d^Direct contact transmission in guinea pig model.

**Table 2:** Amino acid substitutions identified during serial passages of avian H9N2 virus in guinea pigs.

Genes	Amino acid position	Wild virus	P3	P6	P9	P12	P15
**PB2**	195	D	D	**D/N**^c^	N	N	N
**PB1**	NC^a^		ND^b^	ND			
**PA**	NC		ND	ND			
**HA1**^d^	227	Q	P	P	P	P	P
**HA2**	46	D	D	D	D	D	E
**NP**	434	E	K	K	K	K	K
**NA**	NC		ND	ND			
**M1**	211	Q	Q	Q	Q	**Q/R**	R
**M2**	NC		ND	ND			
**NS**	NC		ND	ND			

^a^NC, no amino acid changes detected between wild virus and P15 virus.

^b^ND, sequencing not done.

^c^Bold letters denotes two residues at particular amino acid position based on data of sequencing profiles.

^d^The amino acid positions of HA1 were as H3 HA numbers.
