# Supplementary material for: Adaptation of H9N2 AIV in guinea pigs enables efficient transmission by direct contact and inefficient transmission by respiratory droplets
Source: Sci Rep. 2015 Nov 10;5:15928. doi: 10.1038/srep15928 (PMC4639850; doi:10.1038/srep15928)

Adaptation of H9N2 AIV in guinea pigs enables efficient transmission by direct contact and inefficient transmission by respiratory droplets

Xiaoyu Sang[1,2,3], Airong Wang[2,4], Jie Ding[2], Huihui Kong[1], Xiaolong Gao[1,2], Lin Li[2,5], Tongjie Chai[4], Yuanguo Li[2], Kun Zhang[2]， Chengyu Wang[2], Zhonghai Wan[2], Geng Huang[2], Tiecheng Wang[2], Na Feng[2,5], Xuexing Zheng[2], Hualei Wang[2], Yongkun Zhao[2], Songtao Yang[2], Jun Qian[2,4,6], Guixue Hu[5*],Yuwei Gao[2,4,6,7*], Xianzhu Xia [1,2,4,6,7*]

[1]Harbin Veterinary Research Institute, Chinese Academy of Agricultural Sciences, Harbin, 150001, China

[2]Key Laboratory of Jilin Province for Zoonosis Prevention and Control, Military Veterinary Research Institute, Academy of Military Medical Sciences, Changchun, 130122, China

[3]College of Animal Science and Veterinary Medicine, Shenyang Agricultural University, Shenyang, 110866, China

[4]College of Animal Science and Veterinary Medicine, Shandong Agricultural University, Tai'an, 271000, China

[5]College of Animal Science and Technology, Jilin Agricultural University, Changchun, 130000, China

[6]Changchun Veterinary Research Institute, Chinese Academy of Agricultural Sciences, Changchun, 130122, China

[7]Jiangsu Co-innovation Center for Prevention and Control of Important Animal Infectious Diseases and Zoonoses, Yangzhou, 225000, China

***Corresponding author.** Guixue Hu, huguixue901103@163.com, Yuwei Gao,

gaoyuwei@gmail.com, and Xianzhu Xia, 13944855500@163.com.

Figure S1. Transmission of guinea pig-adapted H9N2 AIV by direct contact. Direct contact-mediated transmission of guinea pig-adapted H9N2 virus was assessed using viruses generated after 9 serial passages (P9, panel a), 12 serial passages (P12, panel b) and 15 serial passages (P15, panel c). Each line represents the virus titer for an individual animal. The dashed lines indicate the lower limit of detection.

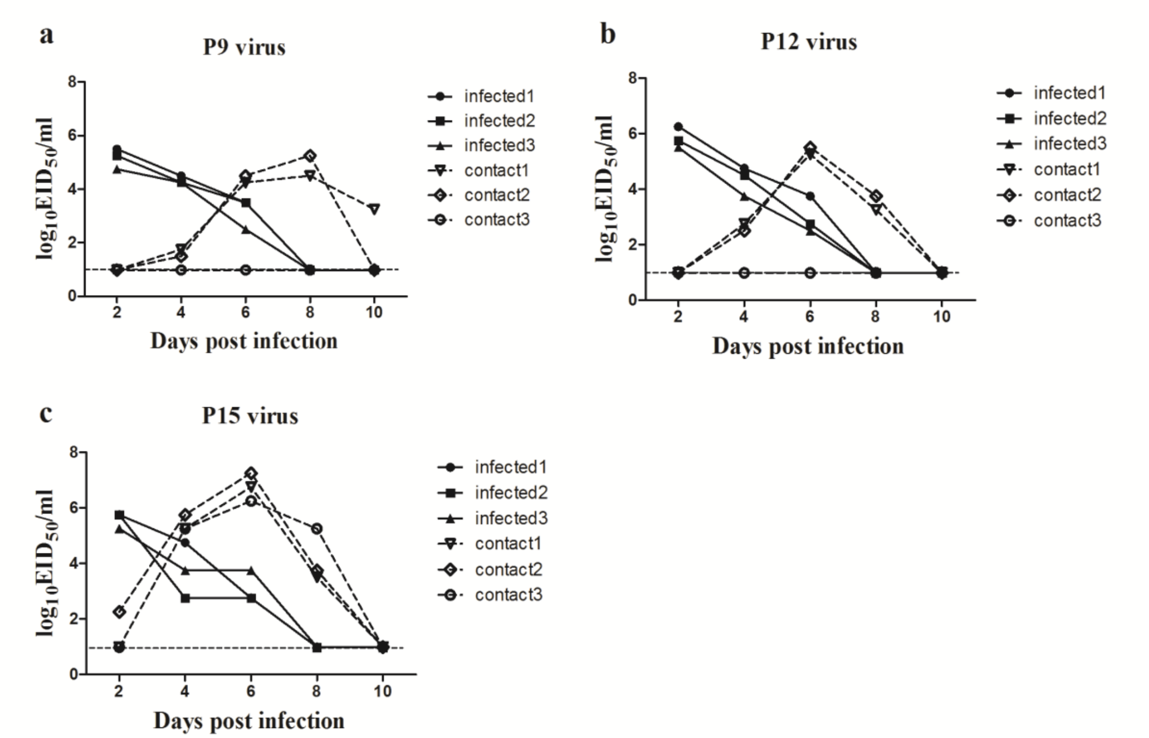

Supplement: Supplementary Information [file srep15928-s1.pdf]
